# Custom Massive Allograft in a Case of Pelvic Bone Tumour: Simulation of Processing with Computerised Numerical Control vs. Robotic Machining

**DOI:** 10.3390/jcm11102781

**Published:** 2022-05-15

**Authors:** Leonardo Vivarelli, Marco Govoni, Dario Attala, Carmine Zoccali, Roberto Biagini, Dante Dallari

**Affiliations:** 1 Reconstructive Orthopaedic Surgery and Innovative Techniques—Musculoskeletal Tissue Bank, IRCCS Istituto Ortopedico Rizzoli, 40136 Bologna, Italy; dante.dallari@ior.it; 2 Department of Oncological Orthopaedics—Musculoskeletal Tissue Bank, IRCCS—Regina Elena National Cancer Institute, 00144 Rome, Italy; dario.attala@ifo.it; 3 Department of Anatomical, Histological, Forensic Medicine and Orthopaedic Science, University of Rome, Piazzale Aldo Moro 5, 00185 Rome, Italy; carmine.zoccali@uniroma1.it; 4 Department of Oncological Orthopaedics, IRCCS—Regina Elena National Cancer Institute, 00144 Rome, Italy; roberto.biagini@ifo.it

**Keywords:** custom allograft, pelvic tumour, massive allograft, tumour reconstruction, bone machining, GMP manufacturing, simulation, virtual planning

## Abstract

The use of massive bone allografts after the resection of bone tumours is still a challenging process. However, to overcome some issues related to the processing procedures and guarantee the best three-dimensional matching between donor and recipient, some tissue banks have developed a virtual tissue database based on the scanning of the available allografts for using their 3D shape during virtual surgical planning (VSP) procedures. To promote the use of future VSP bone-shaping protocols useful for machining applications within a cleanroom environment, in our work, we simulate a massive bone allograft machining with two different machines: a four-axes (computer numerical control, CNC) vs. a five-axes (robot) milling machine. The allograft design was based on a real case of allograft reconstruction after pelvic tumour resection and obtained with 3D Slicer and Rhinoceros software. Machining simulations were performed with RhinoCAM and graphically and mathematically analysed with CloudCompare and R, respectively. In this case, the geometrical differences of the allograft design are not clinically relevant; however, the mathematical analysis showed that the robot performed better than the four-axes machine. The proof-of-concept presented here paves the way towards massive bone allograft cleanroom machining. Nevertheless, further studies, such as the simulation of different types of allografts and real machining on massive bone allografts, are needed.

## 1. Introduction

The limb-saving techniques concerning the reconstruction of massive bone defects after tumour resection remain a challenge for orthopaedic oncologists, especially for issues related to long-term function and durable fixation. Up to the present, several treatment options for bone tumours at diaphyseal/meta-diaphyseal sites of long bones, such as massive allografts, autografts, extracorporeal tumour sterilization or devitalization, segmental transport, endo-prosthetic spacer, or cementoplasty, have been available [1,2,3]. Reconstructions for pelvic sarcomas are even more challenging, since the nononcologic complication and reoperation rates are remarkably high [4]. Generally, the tumour location and the type of complication appear to be related. For instance, nerve damage and bowel injury are notable in an ilio-sacral-type tumour, while bladder injury is common for an ischiopubic one. Moreover, infections remain the most relevant issue to be addressed, affecting between 20% and 80% of oncological patients. Further critical factors, such as older age, prolonged operative time, and poor flap viability may promote microbial contamination, especially in interventions of reconstruction after resection, where an external hemipelvectomy is sometimes needed to eradicate the infection [5].

Among the reconstruction techniques of pelvic bone tumour resections, structural bone allografts (SBAs) have been used over the years, alone or combined with endoprostheses, also achieving satisfactory clinical results thanks to the concomitant application of neoadjuvant chemotherapy, radiotherapy, and targeted drugs [6]. As known, the major advantages of bone harvested from allogeneic sources include its availability in various shapes and sizes and no donor site morbidity. SBAs are procured from cadaver donors collected from several anatomical sites, such as the pelvis, ribs, fibula, humerus, femur, and tibia, and processed by manual or machining cutting techniques to meet the specific dimensional needs planned by surgeons.

Nevertheless, although SBAs are a reasonable choice being their main advantages, the morphologic similarity, and the possibility of osteointegration to the host bone, they exhibit some drawbacks mainly related to the need to have bone bank technology available, as well as a good tissue stock to allow the selection of a piece that matches optimally with the anatomy of the recipient.

Therefore, to guarantee the best three-dimensional matching between donor and recipient, some tissue banks have developed a virtual tissue database based on the scanning of the available allografts by computed tomography (CT) and using their 3D shape during virtual surgical planning procedures (VSP). Specifically, VSP is an emerging and disruptive technology that allows a precise definition of the resection margin together with the 3D shape of the resulting bone defect. Accordingly, VSP also allows the 3D design and 3D printing of patient-specific instruments (PSIs)—e.g., cutting guides—and custom endo-prosthesis, as well as the 3D design of the desired allograft and related graft-specific instruments (GSIs)—e.g., allograft cutting guides [7,8,9,10,11].

Therefore, VSP allows the accurate selection of the most appropriate tissue and the modification of the planning for the selected allograft, as well as the design of GSIs [12,13]. Nevertheless, it is worth noting that, although the shaping of the allograft during the surgery with GSIs leads to good outcomes in terms of dimensional precision, having a pre-shaped allograft represents a decrease in surgical time, as well as a reduction of the infection risk, especially when the shaping is performed in a cleanroom environment.

The accredited public non-profit Musculoskeletal Tissue Bank (hereafter BTM) of IRCCS Istituto Ortopedico Rizzoli (Bologna, Italy; EU TE code: IT000096) performs the harvesting, processing in a GMP class A environment, and distribution of bony and tendinous allografts. The processing of such allografts is performed manually or with a four-axes computerised numerical control (CNC) machine specifically designed for cleanrooms [14]. Moreover, in the past few years, BTM has developed a prototype of a cleanroom milling machine equipped with an industrial robotic arm to perform more complex machining operations on bone allografts.

Therefore, to pave the way for future VSP bone-shaping protocols useful for machining applications within a GMP class A environment, here, we show the machining differences between a four-axes CNC and a robot milling machine resulted from designing a custom allograft from a real case of reconstruction after resection of a pelvic tumour.

Hence, this study opens up future outlooks on the potential application of computerised machined customised massive allografts in orthopaedics and orthopaedic oncology by a theoretical and mathematical approach. Lastly, the information achieved by this study might be applied for 3D scans and/or CT reconstruction analyses of complex tissue geometries.

## 2. Materials and Methods

To simulate via software the processing of a massive bone allograft, a real case of bone tumour resection and subsequent reconstruction with a massive bone allograft was selected. Specifically, a case classified as area 1 according to Enneking et al. [15], and, marginally, 4 and 2, was selected to represent a standard case of complex reconstruction.

**Figure 1:**
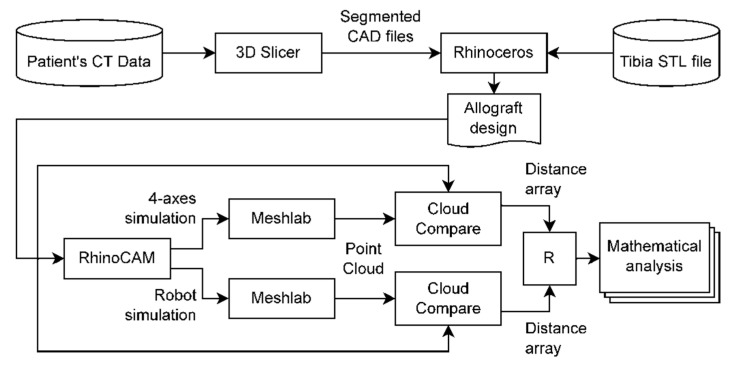
Flowchart of the data and software used in the simulation protocols. (CT: computer tomography; CAD: computer aided design; STL: stereolithography; CAM: computer aided manufacturing).

After the patient’s surgery, CT data from the first post-operative check-up were anonymised and used to perform simulations and relative analyses with several software packages according to the flowchart displayed in Figure 1.

### 2.1. Surgery

The surgery was performed at Istituti Fisioterapici Ospitalieri (Rome, Italy) and was carried out in two steps, the first preparatory (urological, vascular, and orthopaedic parts) and the second for reconstruction.

The first step of preparatory surgery was performed as follows:

1. Urological surgery: preparation of the operating field, insertion of a cystoscope, and placement in the left ureter of a double J stent.
2. General surgery: preparation of the operative field, median xipho-pubic incision, and laparotomy. The intestinal loops were explored, and the ileum was identified. Temporary ileostomy was performed on the right side; the rectum was mobilised by anterior opening and towards the right side, exposing the anterior surface of the sacrum.
3. Vascular Surgery: the left ureter, the common iliac external and internal vascular bundles, and the first branch of the nerve, as well as all tumour-relevant connections, were identified and isolated. The most terminal portion of the vena cava and aorta was mobilised laterally, exposing the anterior aspects of L4-L5-S1. A dissection of the terminal branches of the ileo-lumbar artery present at the height of the L4 vertebra was performed.
4. Orthopaedic Surgery: left partial sagittal osteotomy of L5 and S1 was performed, and haemostatic bone wax was applied; Goretex Dual Mesh sheet was positioned as a divider between the previously isolated neurovascular bundles, the left ureter, and the tumour mass.
5. Repeated washing, revision of haemostasis, suturing for floors, skin in metal clips, sterile dressing, and affixing of an ostomy plate were performed.

The second step of the reconstructive surgical time was performed as follows:

1. Patient in right lateral decubitus; pulse oximeter on the first toe of the left foot. Preparation of the operating field with disposable drapes.
2. Resection: Enneking-type incision was performed, starting from the iliac crest and up to the ischial notch, including the scar from the previous surgery. The posterior portion of the disease was skeletonised, leaving a thin layer of healthy tissue covering the lesion until the sciatic notch was visible. Above, the muscles of the abdominal wall were dissected, and the iliac muscle was detached from the internal side of the disease. Medially, the psoas muscle was spread, along with the femoral nerve. Anteriorly, the origin of the sartorius muscle was detached from the anterior superior iliac spine, the tendon was joined by the anterior-inferior iliac spine, and the joint capsule was visualised. The landmark was positioned in the most anterior portion of the iliac wing, and the navigator was set. The left paravertebral and parasacral groove was skeletonised from L4 to S3. Guided by the navigator, osteotomy of the left sacral wing and the left transverse process of L5 were performed with the aid of chisels and ultrasonic saws. Supracetabular osteotomy was performed, and the operative piece was removed after completion of the section of the soft parts.
3. Reconstruction: vertebral screws were placed in the left pedicle L5 and the left ischium. The tibial allograft from Musculoskeletal Tissue Bank of IRCCS—Regina Elena National Cancer Institute (Roma, Italy; EU TE code: IT000968) was modelled and positioned on the residual portion of the acetabular roof and the sacrum and was fixed with a screw to the ileum and a screw to the vertebral body S1; finally, the bar was assembled and the nuts tightened. Satisfactory fluoroscopic control, multiple washes, culture swab, drainage placement, layered suture, stapled skin, and dressing were performed. The surgery was performed with the aid of 2.5× microsurgical loops.

### 2.2. 3D Slicer—CT Segmentation and 3D Reconstruction

CT scans were performed at Istituti Fisioterapici Ospitalieri (Rome, Italy). All images were acquired using an Optima CT660 CT scanner (GE Healthcare, Milwaukee, WI, USA). The following parameters were used: 120 kVp; 40 mm beam collimation; 2.5 mm image thickness; 310–410 mm reconstruction field of view; 500 mA (maximum); 1 s rotation time.

Optiray 350 (Guerbet, Villepinte, France) intravenous contrast agent was used (100 mL) with the patient in a supine position with arms raised. The field of view extended from the mid-cervical spine to the mid-femoral area. Imaging of the chest, abdomen, and pelvis was performed in the portal phase. Soft tissue, lung, and bone reconstructions were undertaken with appropriate windowing and kernels with ASIR technology.

Digital Imaging and Communications in Medicine (DICOM) data were imported into the software 3D Slicer (version 4.11.20200930, http://www.slicer.org, accessed on 28 February 2022) [16], selecting the sequence related to bone tissues. Through the Module “Segment editor”, after creating a new segmentation set and the first segment, the tool “Threshold” was selected and then defining the range 159.95–3071.00 for masking. Multiple segments, such as the right and left femur, right and left hemipelvis, sacrum, L5–L3 vertebrae, allograft, and bars (including screws), were defined for the post-operative CT. Selecting the “Paint” tool, with the “Sphere brush” option enabled, small points or areas inside bones, bars, and screws were manually defined for each segment, from about 5% up to 40% of the real volume of each segment. Points and small areas were distributed along the volume of each segment, especially near joints, to obtain the best result in the next passages. After that, the “Grow from seeds” tool was selected for a rapid segmentation, setting the “Seed locality” parameter to 10, with the “Intensity range” masking activated. As the last automatic passage, the tool “Islands” was selected, and then, the option “Remove small islands” with 100 voxels as the “Minimum size” was applied. Errors due to mismatching and residual artefacts were corrected manually for the entire allograft segment and the bone–allograft interface of the left hemipelvis, sacrum, and L5 vertebra, since the purpose of this study is the simulation of the computer-defined machining of an allograft similar to the implanted one. The segmentation procedure described here was performed by an engineer and lasted approximately four hours due to the artifacts produced by the presence of metal elements—i.e., bars and screw.

As a final step, all the segments were exported as STL files through the function “Export to files” in the module “Segmentations”.

### 2.3. Rhinoceros—Setting CAD Files for Machining Simulation

**Figure 2:**
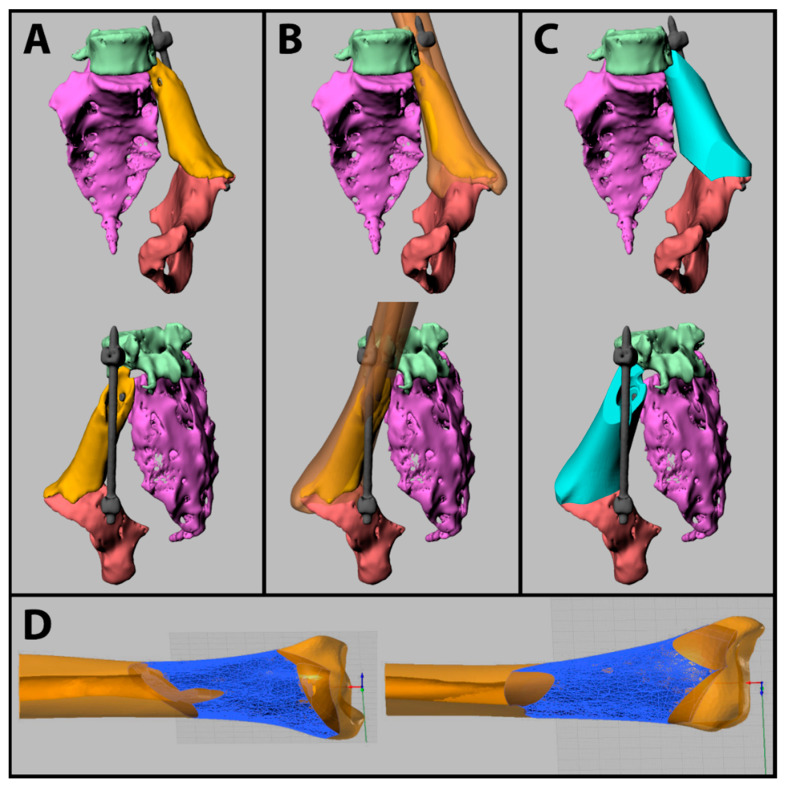
(**A**) Segmented files as imported from 3D Slicer; implanted allograft (yellow); resected hemipelvis (red); resected sacrum (purple); L5 vertebra (green); bars and screws (grey). (**B**) Overlap of the selected allograft model (brown). (**C**) Designed allograft (light blue) in place. (**D**) Designed allograft (blue) vs. allograft model (brown).

Five separate STL files were imported in the software Rhinoceros ver. 4 (www.rhino3d.com, accessed on 28 February 2022), as displayed in Figure 2A: allograft; resected hemipelvis; resected sacrum; L5 vertebra; bars and screws. Additionally, an STL file of a left tibia from BTM allograft archives was imported to simulate the manual matching of an available allograft.

The tibia model was split into two parts, and the distal portion was selected as an allograft model due to more similar dimensions compared with the proximal portion; then, its position and orientation were manually adjusted to obtain the best matching, as displayed in Figure 2B.

Then, the allograft model was shaped as similar as possible to the implanted allograft to simulate the 3D planning of the surgical operation, as displayed in Figure 2C. Multiple cuts were performed on the allograft model using the tool “Meshbooleansplit” with planar and curve surfaces to shape the allograft model and cylindrical cuts to create holes for screws, bars, and the spinal nerve canal. In this virtual planning, to intraoperatively choose which kind of screws to use and where to fix them, surgeons have not required pilot holes for osteosynthesis screws. On the other hand, they have requested the design of a circle engraving inside the medullary canal, as an exact indication of where to fix the screw for the allograft–vertebra interface.

Finally, the designed allograft and the allograft model were saved in a separate Rhinoceros file to set their position and orientation for the machining simulations. Then, the allograft model was exported as a STL file to be used as stock material in machining simulations, thus maintaining its position and orientation. Comparison of the designed allograft and the allograft model used as stock material are shown in Figure 2D.

### 2.4. RhinoCAM—Machining Simulations

The planning of machining trajectories was performed by an engineer using Rhinoceros by the Rhino-CAM 2 plug-in (MecSoft Corporation, Irvine, CA, USA). Two different simulations were performed by the same operator to generate machining trajectories for two different milling machines designed for cleanroom environments and located at BTM: a 4-axes CNC milling machine (Bright model, Delta Macchine, Rieti, Italy), and a robot prototype (RP) milling machine (Lamipress, Bologna, Italy) equipped with an industrial robotic arm (TX-60 CR model, Staubli Italia, Carate Brianza, Italy).

**Figure 3:**
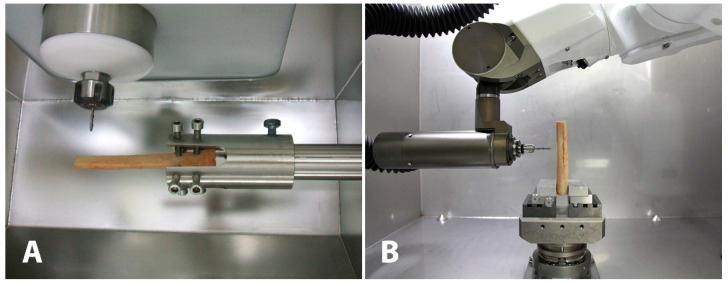
(**A**) Four-axes CNC (computer numerical control) milling machine. (**B**) Robot prototype milling machine.

The simulation of the tissue processing was performed with the related function of the RhinoCAM plug-in as a 4- and 5-axes milling machine for CNC and RP machines, respectively. The industrial robot shown in Figure 3B is a 6 degrees of freedom (DoF) machine, but the sixth axis can be considered as overlapping with the milling tool axis. Thus, it is more appropriate to refer to it as a 5-axes machine. Specifically, three axes are for spatial translation of the tool, the fourth and the fifth axes are for its orientation.

To study the differences between the simulation of tissue processing using the abovementioned machines, identical machining steps were carried out in both simulations, while varying tool path trajectories only in terms of geometry and tool direction. To perform a simulation as very similar as a real bone machining, the milling parameters were based on the experience of the BTM [14]. Specifically, to avoid multiple passages that could lead to bone overheating and long machining time, the distance of the parallel toolpath was set to 25% of the tool diameter.

Since the clamping of the allograft was planned to be on the diaphyseal side, the machining steps were planned as listed in Table 1.

The engraving operation (step 6) was planned with a 2-mm diameter ball mill, since using a 4-mm ball mill would have caused a too-wide furrow even more difficult to machine inside the medullary canal. Machining differences are further presented in Section 3.1.

Once the tool path trajectories were defined, both machining simulations were performed using the functions in the “Simulation” tab of RhinoCAM. Finally, the results were exported through the “Export to STL” tool, imported to a new Rhinoceros file, cleaned from each residual portion, and saved as STL files.

### 2.5. Meshlab—Point Cloud Generation

To mathematically analyse the geometrical differences between the results of each simulation and the designed allograft, a denser point cloud was necessary for the first ones. To this aim, the Meshlab software packages were used (Meshlab 64-bit v2020.12, www.meshlab.net, accessed on 28 February 2022, Visual Computing Lab, ISTI—CNR, Pisa, Italy) [17].

As a first step, the “Poisson-disk Sampling” filter was used [18] with the following parameters: 0.1 as the “Explicit Radius” world unit; 20 as the “MonteCarlo Oversampling”; “Refine Existing Samples” option checked; “Best Sample Heuristic” option checked; 10 as the “Best Sample Pool Size”; 1 as the “Radius Variance”. As a result, a point cloud of almost one million points uniformly distributed on the surface was generated for each simulation. However, to better visualise the point cloud, more steps are needed to generate a mesh.

As a second step, the “Compute normal for point sets” filter was applied, with 10 as the “Neighbour number” and 0 as “Smooth Iteration”.

As a third step, the “Surface Reconstruction: Ball Pivoting” filter was used [19], with 0.15 as the “Pivoting Ball” radius, 20% as the “Clustering radius”, and 90 degrees as the “Angle Threshold”.

As for the final step, the “Invert Faces Orientation” filter was applied with the “Force Flip” option enabled.

### 2.6. CloudCompare—Computation and Visualisation of Points to Mesh Distances

The meshes generated in the previous passage and the one representing the designed allograft were imported to the software CloudCompare (version 2.11.3, www.cloudcompare.org, accessed on 28 February 2022) to compute the distances between the points of the simulation and the surface of the design.

To this aim, once one of the two simulations and the designed allograft were opened, the item “Mesh” was selected for each file; then, the tool “Cloud/Mesh Dist” was applied from the menu “Tools > Distances”. After computing the maximum distance with the tab “Approximate distances”, the following parameters were set in the “General parameters” tab: “AUTO” as “Octree level”; 1 as maximum distance; signed distances checked; multi-threaded checked.

To better highlight the geometrical differences, a custom colour map was set through the “Color Scales Manager” in the “Edit > Scalar fields” menu. The custom colour map was set as follows: red at +1 mm; orange at +0.1 mm; green at 0 mm; light blue at −0.1 mm; blue at −1 mm. After setting the colour map, the application was performed selecting the right mesh and modifying some parameters in the properties tab: “Scalar field” in “Colors” and the name of the custom colour map in “Color Scale > Current”.

Finally, to export the array of distances for analysis purposes, for each mesh, the sub-item “vertices” was selected and saved as a CSV file. Then, the CSV files of the two simulations were merged with Microsoft Excel software (Microsoft Corporation, Redmond, WA, USA).

### 2.7. R Software—Data Elaboration

To perform mathematical analyses on the array of distances, R software was used (version 4.1.2 64bit, R Foundation for Statistical Computing, Vienna, Austria, www.r-project.org, accessed on 28 February 2022) [20].

The R code used to analyse and display data is available as Supplementary Materials File S1. The two arrays were separately analysed, and each array was divided into two groups, positive and negative values, to highlight where the simulated machining tool left in place extra stock material or excessively subtracted an amount of it, respectively.

Since the designed allograft required machining of the distal and proximal portions of the allograft model, the central portion was preserved. Thus, the points corresponding to the central part of the two simulations presented distance values near zero. Hence, to characterise the most significant values of positive and negative distances, a cut-off value of 0.1 mm was used to filter data: positive range from +0.1 mm to 1 mm; negative range from −1 mm to −0.1 mm.

The obtained data were analysed with the summary function and then displayed using the ggplot2 library [21] to analyse the distribution of the values: minimum value, first quartile, median, mean, third quartile, maximum value. Additionally, the sum of all values was also examined, since the distribution of points on the surface allows the use of distances for computing a volumetric comparison between simulations.

## 3. Results

This section shows the graphical and mathematical results, obtained with the methods described in Section 2.6 and Section 2.7, respectively.

### 3.1. CloudCompare Analysis

**Figure 4:**
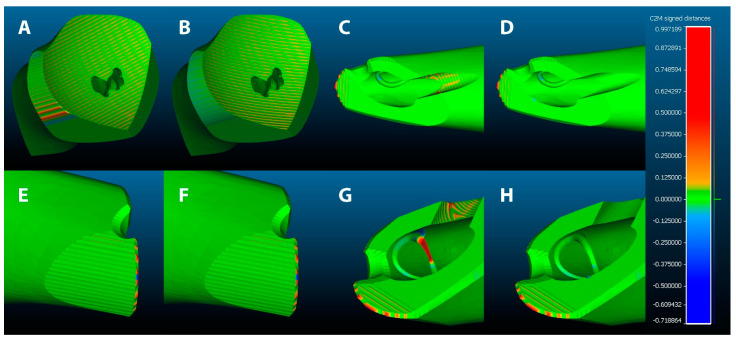
CloudCompare analysis. (**A**,**C**,**E**,**G**) Four-axes simulation. (**B**,**D**,**F**,**H**) Robot simulation. Custom scalar field: red at +1 mm; orange at +0.1 mm; green at 0 mm; light blue at −0.1 mm; blue at −1 mm.

The graphical analysis is shown in Figure 4. Different view angles of the two machined allografts are paired for a more accurate comparison. The left side of each pair is an image from the four-axes simulation, while the right side is from the five-axes simulation.

Red areas represent extra stock material—i.e., where the simulated tool did not sufficiently remove material—and blue areas represent an excessive removal of material—i.e., where the simulated tool removed too much material. On the other hand, the green colour represents areas with no significant differences between the design and the simulation—i.e., non-machined areas or correctly machined ones.

Considering the machining phases as reported in Table 1, the comparison of step 1 is shown in Figure 4A,B. Specifically, the two planar faces on the bottom and the right side of the two images show no differences; the concave face, correspondent to the allograft–pelvis interface, presents different directions of tool trajectory but the same machining strategy; thus, the volumetric differences are not relevant; the curve lateral surface, on the left side of the two images, presents a substantial difference, since the five-axes simulation allowed a proper orientation of the tool; indeed, the four-axes simulation presents visible red areas.

The differences in the bar housing machining (step 2) are shown in Figure 4C,D on the left side and Figure 4G,H on the top right corner. Moreover, as for the curve surface described above, in the five-axes simulation, the ability to orient the tool in two directions guarantees a better result.

The allograft–vertebra interface (step 4), visible in Figure 4E,F, shows no difference, due to the machining operations being the same, because the angulation of the surface does not allow proper orientation of the tool without a collision between the tool holder and the allograft. For the same reason, the machining of the spinal nerve canal (step 3) presents no differences, as shown in Figure 4C,D in the top left corner, in Figure 4E,F in the top right corner, and in Figure 4G,H on the left side.

The machining trajectory of the diaphyseal diagonal plane (step 5) was the same in both simulations, since the results in the four-axes simulation were satisfactory. The results of this phase are clearly visible as green areas in Figure 4C,D,G,H.

The machining trajectory of intramedullary circle engraving (step 6) was the most critical part of this allograft design, as shown in the central portion of Figure 4G,H. For the four-axes simulation shown in Figure 4G, the ability to orient the tool on a single axis has led to the presence of an undercut—i.e., an area that cannot be reached by the tool—that is clearly visible as the red segment. Conversely, in the five-axes simulation shown in Figure 4H, the tool was able to completely change its orientation; thus, the machining operation was correctly completed.

Finally, the last machining trajectory for allograft detachment (step 7) was repeated identically in both simulations, as shown on the bottom left corner of Figure 4G,H, and on the left side of Figure 4C,D. This step has been programmed as rough machining since the weight of the allograft can break off the thin residual support at any time. Therefore, the detaching procedure of the allograft from the clamped portion must be a simple operation, planning a final manual smoothing of rough corners.

### 3.2. R Analysis

Simple data extraction of the statistical characteristics of the distances arrays is shown in Table 2. Specifically, a quartiles analysis has been performed, as well as a length, mean, and a sum comparison. As specified in Section 2.7, each array was subdivided into positive and negative values, with a 0.1 cut off.

The quartile analysis provides a summary of data distribution by ordering (i) values from smallest to largest and extracting the central value (median), (ii) the value between smallest and the median correspondent to 25% (first quartile), and (iii) the value between median and the largest correspondent to 75% (third quartile).

Regarding positive values, corresponding to residual extra stock material, the first noteworthy value is the length of arrays. In the four-axes simulation, there are 8866 points above 0.1 mm, while, in the five-axes simulation, there are 3425 points, less than a half.

In the quartile analysis of the positive values, the minimum is equal to 0.1 for both arrays due to the cut-off value, and all other values of the five-axes array are lower than four-axes ones; in particular, the maximum value is about 25% lower (0.7383 vs. 0.9972), but the most important differences are in the median and third quartile (median: 0.1170 vs. 0.1719; third quartile: 0.1438 vs. 0.2683), suggesting that the distribution of the five-axes positive points is more concentrated towards 0.1 mm.

The mean value—i.e., average obtained by dividing the sum of values by their count—is coherent with the quartile analysis: 0.1483 for the four-axes and 0.2218 for the five-axes. It is worth noting that, while, for the four-axes simulation, the mean value is between the median and the third quartile, for the five-axes, the mean is slightly higher than the third quartile, suggesting the presence of a long tail of outliers—i.e., values lying outside the overall pattern of distribution [22].

Regarding the sum of all values, since the distribution of points on the allograft surface has been structured as uniform, each point can be considered as an arbitrary area unit; thus, the sum can be considered as the volumetric analysis. Coherently with the previously reported data, the sum of the four-axes values is about four-fold higher than the five-axes ones (1966.052 vs. 507.8525), highlighting whether the four-axes machining left much more excess material.

Regarding the negative values, corresponding to excessive removal of material, the length of arrays is considerably lower compared to positive arrays, which is an indication of the machining trajectory accuracy. Moreover, as well as for positive points, the number of four-axes values is higher than five-axes, even nine-fold higher (1149 vs. 139).

The quartile analysis of the negative values shows a similar value for the maximum due to the cut-off threshold, while the minimum value is identical, suggesting that some of the duplicated trajectories led to the same results.

Since we are evaluating the negative values, the other values must be analysed, starting from the highest (shortest distance from zero) to the lowest (greatest distance). The median and the third quartile values of the five-axes simulation are higher than the four-axes values (median: −0.1983 vs. −0.2367; third quartile: −0.1270 vs. −0.1604), suggesting a lower material removal for five-axes, but conversely, the first quartile is lower for the five-axes (−0.4730 vs. −0.3293); this detail is also emerging in the mean values, slightly lower for the five-axes simulation (−0.3067 vs. −0.2549), suggesting a sparser distribution of the five-axes values lying below the median.

However, the sum of the negative values shows that the excessive removal of material is seven-fold lower for the five-axes simulation (−42.63529 vs. −292.922).

**Figure 5:**
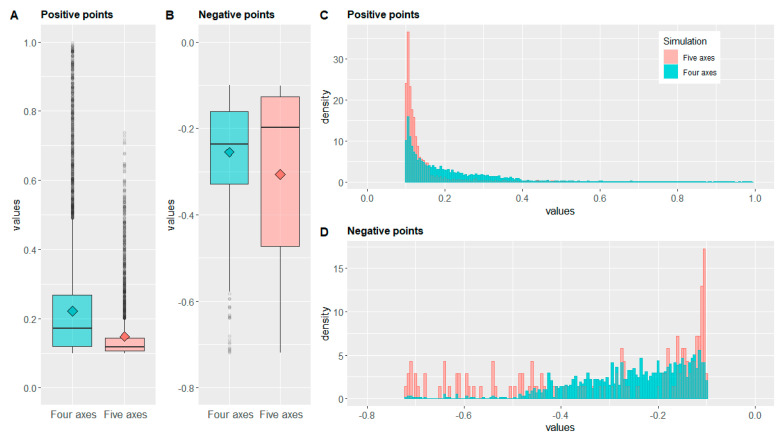
R plot of analysed data, green: the four axes CNC (computer numerical control) machine simulation and red: the five axes Robot simulation. (**A**,**B**) Boxplot comparison of the positive and negative values, respectively (lower line: values from the minimum to the first quartile, box: values from the first to the third quartile, horizontal line: median, square: mean, upper line: values from the third quartile to the maximum, and grey points: outliers). (**C**,**D**) Histogram comparison of positive and negative values respectively (bar width: 0.005).

A graphical plot of analysed data is shown in Figure 5. Specifically, Figure 5A,B represents a boxplot—i.e., quartiles analysis—and mean value of positive and negative points, respectively. Figure 5C,D report histograms of positive and negative points, respectively, to visually analyse the data distribution.

Coherently with values reported in Table 2, Figure 5A shows a more evident proximity of all values to 0.1 for five-axes simulation. However, both simulation presents a long tail of outliers (grey points), but for five-axes, the tail tends to be sparser with the increase of the values. It is worth noting the differences in the box dimensions, as well as for the maximum values.

Boxplots of negative points, as shown in Figure 5B, must be read from top to bottom. According to the description reported above, it is evident that, for the five-axes simulation, the median and the third quartile were closest to −0.1 compared to the four-axes simulation; thus, half of the values—i.e., from the median to maximum value—presents a lower distance from zero. However, the first quartile of the five-axes simulation is significantly lower than four-axes, as well as for the mean value, highlighting a sparser distribution of points; indeed, the lower side of the box is larger for the five-axes simulation.

Such analyses are confirmed in the histograms shown in Figure 5C,D. Specifically, Figure 5C shows positive points where it is clearly visible that most five-axes values are in the 0.1 to 0.2 range, while the distribution of the values for the four-axes simulation is wider.

The abovementioned considerations regarding the negative points can be explained by analysing Figure 5D. As reported in Table 2, where the five-axes negative points are lower in number compared to the four-axes, but as reported in the quartile analysis and Figure 5B, the distribution of values is sparser. Coherently, in Figure 5D, while four-axes points are arranged as an almost continuous distribution, the five-axes points are distributed as small spots (namely, lower in number but sparser). This difference may be due to the circle engraving trajectory, because the complexity of that machining in the five-axes simulation could have generated some points of excessive stock removal, while the overall result remains better.

## 4. Discussion

In orthopaedic surgery there has been a growing development of technologies for precision surgery, starting from planning software [23,24,25,26,27], passing through intraoperative navigation systems [27,28], and up to the recent applications of 3D printing, which involve models for planning [24,29,30], models for plates pre-bending [31,32], and the manufacture of custom prostheses [33,34]. The main purpose of these solutions is twofold: (i) to obtain better clinical outcomes with time savings in the operating room and a related cost reduction and (ii) to decrease the risk of postoperative infections [35,36,37].

Up to the present, regarding bone allograft reconstructions, allograft shaping is usually performed intraoperatively [2,9,38], although having a pre-shaped allograft certainly allows to save surgical time. Unfortunately, although musculoskeletal tissue banks are available worldwide, few of them have the facilities, the expertise, and the know-how to process tissues in a cleanroom environment. Moreover, tissue machining is even less common, limiting this procedure’s availability to intervertebral spacers or small custom grafts [14,39,40,41,42].

The development and investigation of bone processing techniques and machines are characterised by a growing interest in literature. Although the main focus is on orthopaedic surgery robots [28,43,44], some studies investigate the mechanical features of bone processing, such as sawing, milling, and grinding [45,46,47,48,49].

Generally, CNC machines for metal or woodworking have exposed mechanical components that make their complete cleaning difficult. Consequently, it is not easy adapting such machines to the strict requirements of cleanroom environments. Therefore, the use of standard CNC machines for the processing of bone allografts requires the application of less stringent cleaning standards, with the consequent need for final sterilization, which affects the chemical and biomechanical properties of tissues [50,51,52]. Thus, it is mandatory to develop custom CNC machines suitable for controlled contamination environments to avoid the abovementioned critical issues. Accordingly, BTM uses a custom-made four-axes milling machine conceived and manufactured by “Delta Macchine” (Rieti, Italy), which has been used for years for the sterile processing of allografts [14]. Moreover, since there is a growing interest in robot milling machines [53] and industrial robots are characterised by non-exposed mechanical parts to overcome the geometrical limitation of four-axes, BTM has started developing a robot milling machine suitable for cleanrooms through a collaboration with two Italian Companies: Lamipress (Bologna, Italy) and “C.A.T. Progetti” (Bologna, Italy).

To perform a preliminary theoretical validation of the application of the two abovementioned machines to massive bone allograft machining, in this study, the authors simulated through CAM software the machining trajectory of an allograft designed from a real case of reconstruction after resection of a pelvic tumour.

The first part of the process described in Figure 1, regarding CT segmentation, reconstruction, and the design of the allograft, is normally applied for 3D printing of bone models [54,55] or in the design of small allografts but, as it is shown in Figure 2, can be also applied to the design of massive bone allograft. It is worth noting that the used segmentation software, 3D Slicer, is an open-source platform with many tool that make it complex to use; therefore, there are multiple tools and methods to obtain the same result. The segmentation and reconstruction methods here presented took approximately four hours, due to the presence of artifacts; the application of these methods to different patients can lead to a satisfactory 3D reconstruction in a lesser time, albeit the effective time consumption depends on the complexity of CT data, and, mainly, on the operator experience.

After the definition of the design, the described methods continue with the programming of machining trajectories, performed by the same operator in both simulations to avoid possible biases, since machining strategies are highly operator-dependent. Machining trajectories here presented were performed to achieve the best possible result, as if the allograft was to be actually processed and implanted. In fact, machining steps reported in Table 1 in many areas are the same, while the differences are related to the use of RP ability for changing the spatial orientation of the tool. Therefore, machining simulation differences are mainly related to the machine performance.

Trajectories programming must take into account the relation between machining precision features and bone surface roughness—i.e., if an allograft is machined for repairing a small bone defect, the requested precision is higher than for a massive bone area reconstruction. In the selected clinical case, the main purpose was to remove the tumour mass and to restore the structural integrity of the skeletal system in order to allow the patient to walk and to return to normal daily activities. Therefore, high precision for the designed allograft is not mandatory.

Moreover, regarding the interfaces between the allograft and the recipient’s bone, macroscopic rough surface topography can lead to an increase of contact area and friction, which can promote allograft stability. This consideration matches with the machining parameters described in Section 2.4 (namely, the distance of parallel toolpath was set to 25% of the tool diameter to avoid bone overheating and long machining time). Therefore, machining strategies of the two bone–allograft interfaces were similar or identical, as reported in Table 1 and shown in Figure 4A,B,E,F, where the slightly red lines are clearly visible.

The main visually appreciable machining differences shown in Figure 4 are related to (i) the curve lateral surface in Figure 4A,B; (ii) the bar housing in Figure 4 C,D,G,H; and (iii) the circle engraving in Figure 4G,H.

It is worth noting that, for the clinical purpose of this allograft design, the general macroscopic result of four-axes machining is satisfactory, since the differences in the curve lateral surface and the bar housing are not clinically relevant and do not affect the functionality of that area, respectively. On the other hand, the most evident difference is related to the circle engraving inside the medullary canal. Nevertheless, the purpose of the engraving is to recommend to surgeons a position for screw insertion; thus, as shown in Figure 4G, the partial four-axes machining is sufficient.

In this study, although the macroscopic differences between simulations are not clinically relevant, the results achieved with the five-axes simulation aim at serving as a proof of concept of robot machining potential application. The robot machining not only resulted as theoretically feasible, but it has also demonstrated meeting the expectations performing machining trajectories which are not feasible with a four-axes machine, therefore guaranteeing a higher precision.

These considerations are reflected in the mathematical analysis described in Section 2.7 and Section 3.2. Regarding over-machined areas, it is worth noting that the limited number of negative points in both simulations, shown in Table 2 and in Figure 5B,D, as well as the volumetric difference, make this component almost not relevant; thus, a good precision is achieved with both machines. However, for negative points, the five-axes simulation performs better than four-axes.

On the other hand, the most relevant differences regard extra stock material since the number of positive points and their distribution are significant, as shown in Table 2 and Figure 5A,C. Therefore, from this point of view, the five-axes simulation outperforms the four-axes one in all parameters due to the ability to completely change the tool’s spatial orientation by the use of the fifth axis—i.e., the second rotational axis. These data demonstrate a theoretical superiority of robot machining vs. four-axes machining.

However, this study is characterised by some limitations. Firstly, this is a single case simulation to serve as proof of concept, and albeit the selected clinical case is a complex reconstruction procedure, the machining of this type of allograft is not particularly complex; indeed, machining differences between the two simulations are not relevant. Therefore, more cases of pelvic tumour should be analysed, as well as reconstruction of different anatomical sites.

Secondly, this manuscript describes a complex procedure involving 3D CT reconstruction, CAD design, machining programming and simulation, and mesh analyses that require very specific skills not so common in a conventional tissue bank. As a consequence, these complex computational operations were performed by the same operator.

Moreover, the 3D reconstruction described in Section 2.2 is a surface reconstruction that does not consider bone density variation, especially regarding trabecular bone. Therefore, the allograft selection and subsequent design should consider the differences between cortical and trabecular bone.

Lastly, since this study is a theoretical validation, the procedure presented here should be validated with a real massive allograft machining, as many features of real bone machining cannot be simulated—e.g., errors in allograft positioning and clamping, the influence of bone density in the machining results, allograft bending due to tool thrust or geometrical inaccuracies due to machining vibrations.

Considering these limitations, further studies should be carried out in the future. Specifically, to better highlight the characteristics of robot machining compared to four-axes machining, different clinical cases with different type of allografts characterised by more evident undercuts or more complex geometries, such as massive allograft for composite allograft-prostheses reconstructions—i.e., where the complete machining of the medullary canal is mandatory—or hemipelvis machining—i.e., where the complexity of the clamping of allograft will dictate certain machining tool orientations—should be simulated.

Moreover, since many steps of the described procedure are highly operator-dependent, a study with a repetition of the procedure performed by multiple operators should be carried out.

Additionally, a real machining operation of a massive bone allograft should be performed to obtain a complete validation and to achieve the expertise needed to set better planning of machining strategies and trajectories. Such expertise should also be applied in the design procedure, applying a design for the manufacturing approach—i.e., performing the design considering the characteristics of the machine that will be used for manufacturing. It is worth noting that the choice of which machine is more suitable for allograft machining can be based on simulation data, but it should take into account the primary features and actual function of the specific designed allograft, as well as machining trajectory programming time and real complexity and duration of machining procedures.

Finally, since the purpose of allograft reconstruction is patient treatment, following the abovementioned real allograft machining, a clinical trial should be carried out, comparing the use of machined custom allograft with the standard procedure of manual allograft shaping during surgical intervention. Such a clinical trial should evaluate the clinical cost of machining procedure vs. time savings during surgical intervention and, mainly, the assessing of basic activities of daily living in patients who have undergone allograft reconstruction through an ordinal scale index such as the Barthel Index [56] or WHODAS 2.0 [57].

## 5. Conclusions

The robot machining performs better than the four-axes machining due to the ability to completely change the tool’s spatial orientation, albeit in the presented case, the geometrical differences are not clinically relevant. Therefore, although the use of robot milling machines is indicated on a more complex allograft design, characterised by the presence of significant undercuts or elaborate geometries, the proof-of-concept presented here paves the way towards the future challenging application of massive bone allograft cleanroom machining, as well as simulation approaches.

Accordingly, the described methods for the mathematical analyses of geometrical differences can also be applied during the comparison of 3D scans or CT 3D reconstruction of allografts against the allograft design.

Nevertheless, further studies aimed at obtaining a complete validation of the procedure, involving simulation of different allografts—i.e., composite allograft-prostheses reconstructions or hemipelvis—or repetitions of the procedure by independent operators to highlight machining differences and real machining operation of massive bone allografts, are needed.

## Figures and Tables

**Table 1:** Planned machining steps, with definition of the machined area, used tool, and main trajectories differences between CNC (computer numerical control, 4-axes) and RP (robot prototype, 5-axes) simulations.

Step	Machined Area	Tool	Differences
1	Distal portion	Ball mill 4 mm diameter	Similar trajectories, except for lateral curve surface
2	Bars housing	Ball mill 4 mm diameter	Different trajectories
3	Spinal nerve canal	Ball mill 4 mm diameter	Same trajectory
4	Allograft–vertebra interface	Ball mill 4 mm diameter	Same trajectory
5	Part of the diaphyseal portion	Ball mill 4 mm diameter	Same trajectory
6	Intramedullary engraved circle	Ball mill 2 mm diameter	Different trajectories, due to tool orientation ability of RP
7	Rough machining for final detachment	Ball mill 4 mm diameter	Same trajectory

**Table 2:** Length, quartiles, mean, and sum comparison of the distance arrays. Lengths are the number of values, minimum, 1st quartile, median, mean, 3rd quartile, and maximum are in millimetres, and the sum is an arbitrary unit.

Array	Length	Minimum	1st Quartile	Median	Mean	3rd Quartile	Maximum	Sum
Four-axes: positive	8866	0.1000	0.1203	0.1719	0.2218	0.2683	0.9972	1966.052
Five-axes: positive	3425	0.1000	0.1060	0.1170	0.1483	0.1438	0.7383	507.8525
Four-axes: negative	1149	−0.7189	−0.3293	−0.2367	−0.2549	−0.1604	−0.1003	−292.922
Five-axes: negative	139	−0.7189	−0.4730	−0.1983	−0.3067	−0.1270	−0.1009	−42.63529

## Data Availability

The data presented in this study are available on request from the corresponding authors.

## Supplementary Materials

The following supporting information can be downloaded at: https://www.mdpi.com/article/10.3390/jcm11102781/s1, File S1: R script for the analysis and display of data.
